# A longitudinal investigation of the determinants of stroke survivors’ utilisation of a healthy lifestyle for stroke rehabilitation in Australia

**DOI:** 10.1038/s41598-024-78069-z

**Published:** 2024-11-04

**Authors:** Md Sazedur Rahman, Jon Adams, Wenbo Peng, David Sibbritt

**Affiliations:** School of Public Health, Faculty of Health, University of Technology Sydney, Ultimo, NSW 2007 Australia

**Keywords:** Stroke, Healthy lifestyle, Self-management, Rehabilitation, Longitudinal, Public health, Risk factors

## Abstract

This study aimed to determine the longitudinal predictors of lifestyle behaviours among stroke survivors in New South Wales, Australia. This longitudinal study utilised data from the baseline survey (2005–2009) and a sub-study survey (2017) of the 45 and Up Study. Physical activity, alcohol consumption, smoking status, and supplement use were included as dependent variables. Generalised estimating equation models were employed to assess the longitudinal association between the dependent variable and demographic and health status measures. The average age of the participants (n = 576) was 67 (SD = 9) years at baseline and 76 (SD = 9) years at the sub-study survey time, with 54.9% being male. The longitudinal analysis revealed that the likelihood of moderate/high physical activity significantly declined over time and was lower among participants with diabetes, but was higher among those with university education. The likelihood of smoking was significantly higher in females, moderate/high-risk alcohol consumers, and those with depression, but was lower among supplement users. The likelihood of moderate/high-risk alcohol consumption significantly declined with time, and was lower among females, but higher among smokers. The likelihood of supplement use significantly declined over time, but was higher among females and/or those with asthma. Our findings help illustrate that many stroke survivors may benefit from further support in adopting and maintaining a healthy lifestyle as part of their stroke management and long-term rehabilitation, which is crucial to optimising their quality of life and successful secondary stroke prevention.

## Introduction

Stroke is a sudden onset of symptoms of localised neurological dysfunction caused by an interruption in blood flow to the brain^1^. It is a leading cause of adult disability and mortality, and the number of people having a stroke event continues to rise and is predicted to increase significantly in future decades^2,3^. For example, the number of stroke incidents grew by 70% between 1990 and 2019 worldwide, and by 2050, the number of stroke survivors is expected to nearly double (200 million) compared to 2019 (101 million)^3^. From the perspectives of both healthcare and research, it is essential to address the long-term demands of post-stroke individuals^4^.

In 2018, there were approximately 387,000 Australians aged ≥ 15 years (1.3% of the population) who had survived a stroke, with around 39,500 individuals experiencing a stroke each year, resulting in long-term physical and mental disabilities that place a significant burden on their families, communities, and country’s healthcare systems^5^. Common consequences of a stroke include permanent disability, restrictions in motor function, fatigue, pain, falls, dysarthria, aphasia, memory deficits, cognitive impairments, visual problems, social isolation, anxiety, depression, and dependency, among others^6,7^. Moreover, almost 43% of primary stroke survivors are at risk of a second stroke within ten years^8^. The death rate among recurrent stroke survivors is around 41% within the first month after the first recurrent event, which is significantly higher than primary stroke survivors (22%)^8^; therefore, preventing stroke recurrence is an important way to reduce the mortality rate from stroke. Consequently, several international clinical guidelines strongly recommend that stroke survivors adopt self-management strategies, including a healthy lifestyle, as part of their long-term rehabilitation after stroke^9–13^.

Self-management is a widely accepted strategy for reducing the chronic disease burden and supporting people in managing their long-term conditions more efficiently^14^. The term “self-management” is defined as a development process where individuals maintain, monitor, manage, and prevent chronic conditions through the practice of a healthy lifestyle, behaviours and activities, and medical interventions^14^. Self-management has emerged as a potentially useful strategy for stroke rehabilitation in recent years since it enables stroke survivors with long-term management and provides a pathway to maximise stroke recovery, and some recent research has focused on exploring this area of stroke survivorship^15–18^. For example, it has been empirically established that lifestyle modification or maintaining a healthy lifestyle is the most common self-management strategy for stroke rehabilitation^15–18^.

Unhealthy behaviours, such as physical inactivity, inadequate nutrition, excessive alcohol intake, and smoking are major primary and secondary stroke risk factors^19,20^. Stroke survivors are more likely to have low levels of physical activity, most likely due to their disability, fatigue, functional limitations, inaccessible surroundings, lack of motivation, depression, and lack of social support^4,21^. For successful self-management, these stroke-related challenges need to be overcome^4^. Moderate to vigorous physical activity (≥ 150 min/week) is important in stroke rehabilitation, where such activity can improve functional capacity (such as muscle strength, upper limb motor movement, and balance), cardiovascular health, confidence, and quality of life in stroke survivors, as well as reduce their risk of subsequent stroke^4,20,22–24^. Similarly, nutritional supplements, quitting smoking, and consuming less alcohol are also associated with improved health status, mental well-being, physical performance, the prevention of further strokes, brain recovery, and lower nutrition-related diseases and mortality rates among stroke survivors^20,24–29^.

Despite the recent and emerging research on post-stroke self-management, little is known about the adoption and maintenance of healthy lifestyle behaviours among stroke survivors. No large-scale longitudinal study has been undertaken to evaluate the related determinants of a healthy lifestyle among stroke survivors in Australia. To directly address this gap, this study investigated the longitudinal determinants of lifestyle behaviours among stroke survivors in New South Wales (NSW), Australia.

## Methods

### Sample

The data were retrieved from the baseline survey and a sub-study survey of the Sax Institute’s 45 and Up Study conducted in Australia. The baseline survey collected data from ≥ 45 years of age male and female residents of NSW, Australia. The 45 and Up Study details are provided elsewhere^30^. In brief, participants were selected at random from the Services Australia Medicare enrolment database to assure coverage of almost the whole population. To ensure statistical power, ≥ 80 years old people as well as remote and rural inhabitants were oversampled due to their smaller populations. Participants were enrolled in the study between 2005 and 2009 by answering a baseline postal questionnaire and providing informed consent to participate and long-term follow-up. The baseline survey collected data from 267,357 individuals, with an approximate 19% response rate, representing nearly 11% of the population of New South Wales aged 45 and older. Between April and October 2017, a sub-study survey of respondents from this cohort was conducted^17^. The sub-study questionnaire was sent to 1300 participants who stated they had been diagnosed with a stroke on the baseline 45 and Up study survey. Both the baseline and sub-study data included information on social and demographic characteristics; health behaviours (e.g., physical activity, smoking, supplement use, alcohol consumption); general health; and health service utilisation^31^. A total of 576 stroke survivors completed and returned the sub-study questionnaire, with a response rate of 44.3%. The data from these 576 stroke survivors were analysed and reported in this study.

### Outcome variables

The outcome variables for this study were physical activity, alcohol consumption status, smoking status, and supplement use. The measures of outcome variables were as follows:

In the baseline and sub-study questionnaires, physical activity was calculated using questionnaires from the Active Australia Survey^32^, whose reliability and validity have been demonstrated to be acceptable as a self-reported measure of physical activity^33^. Participants reported their weekly frequency and time (hours and minutes) engaged in: (i) continuous walking (W) for at least 10 min; (ii) moderate physical activities (M) (such as social tennis, gentle swimming, gardening/housework); and (iii) vigorous physical activities (V) that caused them to breathe more heavily or puff and pant (such as jogging, cycling, aerobics, competitive tennis). According to the Active Australia Survey, the value of time spent in vigorous physical activity was double that of time spent in lower-intensity physical activity^32^. Therefore, the time of physical activity during the previous week was computed as W + M + 2V, where W, M, and V denote the total amount of time spent on walking, moderate physical activity, and vigorous physical activity, respectively^34,35^. According to the physical activity recommendations in adults, participants were categorised as inactive/sedentary if they engaged in physical activity for < 150 min/week, and moderately/highly active if they engaged in physical activity for ≥ 150 min/week^35^.

The total number of alcoholic drinks was calculated from the item “how many alcoholic drinks do you have each week?” (one drink equals a small glass of wine, middy of beer or nip of spirits). The risk of alcohol consumption was classified as “no/low risk” if the participant consumed ≤ 14 drinks/week, and as “moderate/high risk” if they consumed > 14 drinks/week, in accordance with Australian alcohol guidelines designed to reduce the risk of long-term harm^36^.

Current smokers were identified in the baseline survey using the question “are you a regular smoker now?” In contrast, current smokers during the sub-study period were identified using the question, “how often do you currently smoke cigarettes or any tobacco products?”.

For both the baseline and sub-study periods, supplement usage was identified if any of the following items were used within the previous month: multivitamins, minerals, omega-3, or fish oil.

### Covariates

Covariates included gender (male, female), education (no formal school/school only, trade/apprentice/diploma, university), marital status (single, married/living with a partner, widowed/divorced/separated), body mass index (BMI), hypertension, heart disease, diabetes, depression, anxiety, and asthma.

At baseline and sub-study, self-reported height (m) and weight (kg) were used to determine the BMI (kg/m^2^). According to World Health Organization classifications, BMI was categorised as follows: underweight or normal (< 25.0 kg/m^2^), overweight (25.0–29.9 kg/m^2^), and obese (BMI ≥ 30.0 kg/m^2^)^37^. The presence of hypertension, heart disease, diabetes, depression, anxiety, and asthma were determined from the questions “has a doctor ever told that you have …” [any of these particular conditions] in the baseline questionnaire and “in the past 12 months, have you been diagnosed or treated by a doctor for any of the following disease”, in the sub-study questionnaire.

### Statistical analysis

Bivariate association between a dependent variable and an independent variable was assessed using Chi-square tests. Generalised estimating equation (GEE) models, specifying the binomial family with logit link function and robust standard errors, were employed to assess the longitudinal association between a dependent variable and independent variables. GEE was chosen for its capacity to address within-subject correlations in a repeated measures framework and for delivering robust estimates, making it suitable for our emphasis on population-averaged effects. All variables that returned a p < 0.25 in bivariate analysis were entered into a GEE model and adjusted odds ratios (AORs) were calculated. Backwards stepwise regression was employed to systematically exclude non-significant variables, producing a more parsimonious model, which was especially useful due to the exploratory character of the study and a large number of covariates. We assessed multicollinearity across variables utilising variance inflation factors (VIF), and no significant multicollinearity was identified (VIF < 10). Sensitivity analyses were performed by examining alternative model specifications and adjusting for potential confounding factors. The primary findings remained consistent across various iterations of the model. The statistical software Stata 14.0 was utilised throughout all of the analysis processes. The level of statistical significance for each test was set at p < 0.05.

### Ethical approval

The 45 and Up Study was granted ethical approval by the Human Research Ethics Committee (HREC) of the University of New South Wales. HREC at the University of Technology Sydney permitted the use of the baseline and sub-study datasets from the 45 and Up Study in this study (approval number: ETH19-3442). The participants provided clear written consent to participate and long-term follow-up in the 45 and Up study. All methods were performed in accordance with the relevant guidelines and regulations.

## Results

This study included a total of 576 stroke survivors who had participated in both the baseline and sub-study surveys. Table 1 displays the demographic and health status characteristics of the participants. The majority of the sample was male (54.9%). The average age of the participants was 66.5 (SD = 9.1) years at baseline and 75.8 (SD = 9.1) years at the time of the sub-study survey. Of note, there were some changes over time, where the percentage of smokers increased from baseline (6.9%) to sub-study (10.4%), but the percentage of moderate/high-risk alcohol consumers declined from baseline (17.0%) to sub-study (12.6%). Similarly, moderate/high physical activity (76.4% at baseline and 62.7% in sub-study) and supplements use (43.2% at baseline and 18.1% in sub-study) decreased over time.

**Table 1:** Demographic and health status characteristics of the participants.

Characteristics	Baseline (2005–2009)		Sub-study (2017)	
	Frequency	Percentage	Frequency	Percentage
Gender				
Male	316	54.9	316	54.9
Female	260	45.1	260	45.1
Education				
No formal school/school only	264	46.4	276	48.3
Trade/apprentice/diploma	194	34.1	187	32.8
University	111	19.5	108	18.9
Marital status				
Single	44	07.7	51	9.0
Married/living with partner	402	70.3	359	63.3
Widowed/divorced/separated	126	22.0	157	27.7
Smoking				
No	536	93.1	490	89.6
Yes	40	6.9	57	10.4
Alcohol consumption risk				
None/low	468	83.0	487	87.4
Moderate/high	96	17.0	70	12.6
BMI (kg/m^2^)				
Underweight or normal (< 25.0)	185	34.8	180	35.4
Overweight (25.0–29.9)	223	42.0	195	38.4
Obese (≥ 30.0)	123	23.2	133	26.2
Physical activity				
Inactive/sedentary (< 150 min)	131	23.6	199	37.3
Moderate/high (≥ 150 min)	425	76.4	335	62.7
Hypertension				
No	324	56.3	374	64.9
Yes	252	43.7	202	35.1
Heart disease				
No	460	79.9	438	76.0
Yes	116	20.1	138	24.0
Diabetes				
No	498	86.5	474	82.3
Yes	78	13.5	102	17.7
Depression				
No	431	86.4	519	90.1
Yes	68	13.6	57	9.9
Anxiety				
No	451	90.4	524	91.0
Yes	48	9.6	52	9.0
Asthma				
No	437	87.6	521	90.5
Yes	62	12.4	55	9.5
Supplements use				
No	327	56.8	472	81.9
Yes	249	43.2	104	18.1

Table 2 presents the cross-sectional association between physical activity and demographic and health status characteristics. Statistically significant associations were identified between physical activity and six characteristics at baseline and/or the sub-study. At baseline, a greater percentage of participants who undertook a moderate or high level of physical activity were smokers (p = 0.025), moderate/high-risk alcohol consumers (p = 0.035), had underweight or normal BMI (p = 0.019), and/or did not have asthma (p = 0.029). Similarly, in the sub-study, a greater percentage of participants who undertook a moderate or high level of physical activity were those who did not have diabetes (p = 0.015), had an overweight BMI (p = 0.025), and had university-level education (p = 0.011).

**Table 2:** Association between physical activity and demographic and health status characteristics.

Characteristics	Physical activity					
	Baseline (2005–2009)			Sub-study (2017)		
	Inactive/sedentary	Moderate/high	*p*-value	Inactive/sedentary	Moderate/high	*p*-value
	n (%)	n (%)		n (%)	n (%)	
Gender			0.503			0.883
Male	69 (22.5)	238 (77.5)		108 (37.0)	184 (63.0)	
Female	62 (24.9)	187 (75.1)		91 (37.6)	151 (62.4)	
Education			0.727			0.011
No formal school/school only	60 (23.8)	192 (76.2)		106 (43.6)	137 (56.4)	
Trade/apprentice/diploma	48 (25.4)	141 (74.6)		63 (35.0)	117 (65.0)	
University	23 (21.3)	85 (78.7)		29 (27.4)	77 (72.6)	
Marital status			0.760			0.814
Single	08 (19.0)	34 (81.0)		20 (40.8)	29 (59.2)	
Married/living with partner	93 (23.7)	299 (76.3)		121 (36.4)	211 (63.6)	
Widowed/divorced/Separated	29 (24.6)	89 (75.4)		55 (38.2)	89 (61.8)	
Smoking			0.025 ^F^			0.490
No	128 (24.7)	391 (75.3)		172 (36.7)	297 (63.3)	
Yes	*	> 32 (> 86.5)^A^		22 (41.5)	31 (58.5)	
Alcohol consumption risk			0.035			0.415
None/low	114 (25.2)	338 (74.8)		170 (37.5)	283 (62.5)	
Moderate/high	14 (15.0)	79 (85.0)		21 (32.3)	44 (67.7)	
BMI (kg/m^2^)			0.019			0.025
Underweight or normal	28 (15.7)	150 (84.3)		65 (38.0)	106 (62.0)	
Overweight	56 (25.7)	162 (74.3)		55 (29.7)	130 (70.3)	
Obese	33 (28.0)	85 (72.0)		55 (44.7)	68 (55.3)	
Hypertension			0.801			0.599
No	75 (24.0)	238 (76.0)		122 (36.4)	213 (63.6)	
Yes	56 (23.0)	187 (77.0)		77 (38.7)	122 (61.3)	
Heart disease			0.823			0.296
No	103 (23.4)	338 (76.7)		144 (36.0)	256 (64.0)	
Yes	28 (24.3)	87 (75.7)		55 (41.0)	79 (57.0)	
Diabetes			0.076			0.015
No	107 (22.3)	373 (77.7)		152 (34.9)	284 (65.1)	
Yes	24 (31.6)	52 (68.4)		47 (48.0)	51 (52.0)	
Depression			0.889			0.203
No	94 (22.6)	321 (77.4)		175 (36.4)	306 (63.6)	
Yes	15 (23.4)	49 (76.6)		24 (45.3)	29 (54.7)	
Anxiety			0.535			0.075
No	100 (23.1)	332 (76.9)		175 (36.1)	310 (63.9)	
Yes	09 (19.1)	38 (80.9)		24 (49.0)	25 (51.0)	
Asthma			0.029			0.410
No	89 (21.2)	331 (78.8)		182 (37.8)	299 (62.2)	
Yes	20 (33.9)	39 (66.1)		17 (32.1)	36 (68.0)	
Supplements use			0.446			0.998
No	78 (24.8)	237 (75.2)		161 (37.3)	271 (62.7)	
Yes	53 (22.0)	188 (78.0)		38 (37.2)	64 (62.8)	

*p*-value is obtained using Chi-square test, unless indicated by ^F^ in which case the* p*-value is obtained using Fisher Exact test. *Indicates n < 5 (disclosure of such small numbers in publications presents a potential risk to confidentiality and is prohibited by The Sax Institute’s 45 and Up Study).

^A^The exact number and percentage are not mentioned in order to prevent the identification of small numbers (< 5) within the corresponding data cell.

A longitudinal GEE model was used to determine the most important factors for predicting moderate to high levels of physical activity (Table 3). Participants were 47% less likely to be moderately/highly physically active at the time of the sub-study (AOR = 0.53; 95% CI: 0.42, 0.68; p < 0.001) than the baseline period. Similarly, moderate/high physical activity was 37% lower among participants with diabetes (AOR: 0.63; 95% CI: 0.43, 0.91; p = 0.015) than the non-diabetic participants. Conversely, moderate/high physical activity was 1.60 times higher among participants with university education (AOR = 1.60; 95% CI: 1.07, 2.39; p = 0.022) than the participants with no formal school/school education.

**Table 3:** Output of a Generalized Estimating Equation (GEE) model predicting physical activity.

Variables	Moderate/high physical activity *	
	AOR (95% CI)	*p*-value
Time		
Baseline	1.00	
Sub-study	0.53 (0.42, 0.68)	< 0.001
Education		
No formal school/school only	1.00	
Trade/apprentice/diploma	1.19 (0.87, 1.61)	0.271
University	1.60 (1.07, 2.39)	0.022
Diabetes		
No	1.00	
Yes	0.63 (0.43, 0.91)	0.015

*Reference category: Inactive/Sedentary.

*AOR* adjusted odds ratio.

Table 4 shows the cross-sectional association between smoking status and the selected demographic and health status characteristics. Statistically significant associations were identified between smoking status and seven characteristics at baseline and/or the sub-study. At baseline, a greater percentage of participants who were smokers were female (p = 0.022), consumed alcohol at moderate or high-risk levels (p = 0.001), undertook moderate to high levels of physical activity (p = 0.025), and/or did not have hypertension (p = 0.002). At both the baseline and sub-study periods, a greater percentage of participants who were smokers were single (p < 0.05), had depression (p < 0.05) and/or had anxiety (p < 0.05).

**Table 4:** Association between smoking and demographic and health status characteristics.

Characteristics	Smoking					
	Baseline (2005–2009)			Sub-study (2017)		
	No	Yes	*p*-value	No	Yes	*p*-value
	n (%)	n (%)		n (%)	n (%)	
Gender			0.022			0.069
Male	301 (95.2)	15 (4.7)		277 (91.7)	25 (8.3)	
Female	235 (90.4)	25 (9.6)		213 (86.9)	32 (13.1)	
Education			0.818			0.173
No formal school/school only	245 (92.8)	19 (7.2)		224 (87.8)	31 (12.2)	
Trade/apprentice/diploma	181 (93.3)	13 (6.7)		161 (89.4)	19 (10.6)	
University	105 (94.6)	06 (5.4)		101 (94.4)	06 (5.6)	
Marital status			< 0.001			< 0.001
Single	36 (81.8)	08 (18.2)		34 (70.8)	14 (29.2)	
Married/living with partner	385 (95.8)	17 (4.2)		324 (94.2)	20 (5.8)	
Widowed/divorced/separated	111 (88.1)	15 (11.9)		126 (86.3)	20 (13.7)	
Alcohol consumption risk			0.001			0.056
None/low	443 (94.7)	25 (5.3)		421 (91.3)	40 (8.7)	
Moderate/high	82 (85.4)	14 (14.6)		58 (84.1)	11 (15.9)	
BMI (kg/m^2^)			0.139			0.862
Underweight/normal	167 (90.3)	18 (9.7)		152 (89.9)	17 (10.1)	
Overweight	211 (94.6)	12 (5.4)		174 (91.6)	16 (8.4)	
Obese	117 (95.1)	06 (4.9)		114 (90.5)	12 (9.5)	
Physical activity			0.025 ^F^			0.490
Inactive/sedentary	–	*		172 (88.7)	22 (11.3)	
Moderate/high	–	–		297 (90.6)	31 (9.4)	
Hypertension			0.002			0.915
No	292 (90.1)	32 (9.1)		313 (89.7)	36 (10.3)	
Yes	244 (96.8)	08 (3.2)		177 (89.4)	21 (10.6)	
Heart disease			0.212			0.335
No	425 (92.4)	35 (7.6)		367 (88.9)	46 (11.1)	
Yes	111 (95.7)	05 (4.3)		123 (91.8)	11 (8.2)	
Diabetes			0.636 ^F^			0.908
No	–	–		401 (89.5)	47 (10.5)	
Yes	–	*		89 (89.9)	10 (10.1)	
Depression			0.024			0.047
No	406 (94.2)	25 (5.8)		445 (90.5)	47 (9.5)	
Yes	59 (86.8)	09 (13.2)		45 (81.8)	10 (18.2)	
Anxiety			0.025			0.020
No	424 (94.0)	27 (6.0)		450 (90.5)	47 (9.5)	
Yes	41 (85.4)	07 (14.6)		40 (80.0)	10 (20.0)	
Asthma			0.999 ^F^			0.451
No	–	–		445 (89.9)	50 (10.1)	
Yes	–	*		45 (86.5)	07 (13.5)	
Supplements use			0.080			0.850
No	299 (91.4)	28 (8.6)		399 (89.5)	47 (10.5)	
Yes	237 (95.2)	12 (4.8)		91 (90.1)	10 (9.9)	

*p*-value is obtained using Chi-square test, unless indicated by ^F^ in which case the* p*-value is obtained using Fisher Exact test. *Indicates n < 5 (disclosure of such small numbers in publications presents a potential risk to confidentiality and is prohibited by The Sax Institute’s 45 and Up Study). The numbers and percentages are not mentioned in order to prevent the identification of small numbers (< 5) within the corresponding data cell.

A longitudinal GEE model was used to determine the most important factors for predicting smoking status (Table 5). The model indicates that: female participants were 2.28 times more likely to smoke (AOR = 2.28; 95% CI: 1.23, 4.21; p = 0.009) than male participants; moderate/high-risk alcohol consumers were 2.61 times more likely to smoke (AOR: 2.61; 95% CI: 1.43, 4.76; p = 0.002), than the none/low risky alcohol consumers; and participants with depression were 2.72 times (AOR: 2.72; 95% CI: 1.61, 4.58; p < 0.001) more likely to smoke than the non-depressed participants. Conversely, the odds of smoking was 81% lower among participants who were married/living with a partner (AOR = 0.19; 95% CI: 0.09, 0.40; p < 0.001) compared to the single participants; the odds of smoking was 49% lower among participants with hypertension (AOR: 0.51; 95% CI: 0.33, 0.79; p = 0.003) than the non-hypertensive participants; and the odd of smoking was 40% lower among supplements users (AOR: 0.60; 95% CI: 0.39, 0.92; p = 0.020) than who did not use supplements.

**Table 5:** Output of a Generalized Estimating Equation (GEE) model predicting smoking status.

Characteristics	Smoking status*	
	AOR (95% CI)	*p*-value
Gender		
Male	1.00	
Female	2.28 (1.23, 4.21)	0.009
Marital status		
Single	1.00	
Married/living with partner	0.19 (0.09, 0.40)	< 0.001
Widowed/divorced/separated	0.49 (0.23, 1.04)	0.065
Alcohol consumption risk		
None/low	1.00	
Moderate/high	2.61 (1.43, 4.76)	0.002
Hypertension		
No	1.00	
Yes	0.51 (0.33, 0.79)	0.003
Depression		
No	1.00	
Yes	2.72 (1.61, 4.58)	< 0.001
Supplements		
No	1.00	
Yes	0.60 (0.39, 0.92)	0.020

*Reference category: no smoking.

*AOR* adjusted odds ratio.

Table 6 provides the cross-sectional association between the risk of alcohol consumption and the demographic and health status characteristics. Statistically significant associations were identified between alcohol consumption status and only five characteristics at baseline and/or the sub-study. At baseline, a greater percentage of participants who consumed alcohol at moderate or high-risk levels were smokers (p = 0.001), moderately/highly physically active (p = 0.035); had hypertension (p = 0.025), and/or did not have asthma (p = 0.006). At both the baseline and sub-study periods, a greater percentage of participants who consumed alcohol at moderate or high-risk levels were male (p < 0.001).

**Table 6:** Association between alcohol consumption and demographic and health status characteristics.

Characteristics	Risk of alcohol consumption					
	Baseline (2005–2009)			Sub-study (2017)		
	None/low	Moderate/high	*p*-value	None/low	Moderate/high	*p*-value
	n (%)	n (%)		n (%)	n (%)	
Gender			< 0.001			< 0.001
Male	231 (74.5)	79 (25.5)		249 (81.4)	57 (18.6)	
Female	237 (93.3)	17 (6.7)		238 (94.8)	13 (5.2)	
Education			0.517			0.715
No formal school/school only	221 (84.7)	40 (15.3)		234 (87.6)	33 (12.4)	
Trade/apprentice/diploma	158 (83.6)	31 (16.4)		158 (88.3)	21 (11.7)	
University	87 (79.8)	22 (20.2)		91 (85.1)	16 (14.9)	
Marital status			0.270			0.335
Single	32 (74.4)	11 (25.6)		39 (81.2)	09 (18.8)	
Married/living with partner	332 (84.1)	63 (15.9)		311 (88.6)	40 (11.4)	
Widowed/divorced/separated	100 (82.0)	22 (18.0)		130 (86.7)	20 (13.3)	
Smoking			0.001			0.056
No	443 (84.4)	82 (15.6)		421 (87.9)	58 (12.1)	
Yes	25 (64.1)	14 (35.9)		40 (78.4)	11 (21.6)	
BMI (kg/m^2^)			0.746			0.478
Underweight or normal	152 (84.0)	29 (16.0)		155 (89.1)	19 (10.9)	
Overweight	177 (81.2)	41 (18.8)		161 (85.2)	28 (14.8)	
Obese	100 (83.3)	20 (16.7)		117 (88.6)	15 (11.4)	
Physical activity			0.035			0.415
Inactive/sedentary	114 (89.1)	14 (10.9)		170 (89.0)	21 (11.0)	
Moderate/high	338 (81.1)	79 (18.9)		283 (86.5)	44 (13.5)	
Hypertension			0.025			0.866
No	273 (86.1)	44 (13.9)		315 (87.3)	46 (12.7)	
Yes	195 (79.0)	52 (21.0)		172 (87.8)	24 (12.2)	
Heart disease			0.801			0.168
No	375 (82.8)	78 (17.2)		367 (86.4)	58 (13.6)	
Yes	93 (83.8)	18 (16.2)		120 (90.9)	12 (9.1)	
Diabetes			0.311			0.138
No	401 (82.3)	86 (17.7)		396 (86.5)	62 (13.5)	
Yes	67 (87.0)	10 (13.0)		91 (91.9)	08 (8.1)	
Depression			0.437			0.683
No	349 (82.5)	74 (17.5)		439 (87.6)	62 (12.4)	
Yes	57 (86.4)	09 (13.6)		48 (85.7)	08 (14.3)	
Anxiety			0.739			0.749
No	367 (82.8)	76 (17.2)		444 (87.6)	63 (12.4)	
Yes	39 (84.8)	07 (15.2)		43 (86.0)	07 (14.0)	
Asthma			0.006 ^F^			0.926
No	–	–		440 (87.5)	63 (12.5)	
Yes	–	*		47 (87.1)	07 (12.9)	
Supplements use			0.089			0.726
No	258 (80.6)	62 (19.4)		395 (87.2)	58 (12.8)	
Yes	210 (86.1)	34 (13.9)		92 (88.5)	12 (11.5)	

*p*-value is obtained using Chi-square test, unless indicated by ^F^ in which case the* p*-value is obtained using Fisher Exact test. *Indicates n < 5 (disclosure of such small numbers in publications presents a potential risk to confidentiality and is prohibited by The Sax Institute’s 45 and Up Study). -The numbers and exact percentages are not mentioned in order to prevent the identification of small numbers (< 5) within the corresponding data cell.

A longitudinal GEE model was used to determine the most important factors for predicting alcohol consumption status (Table 7). The model shows that participants were 31% less likely to engage in moderate/high-risk alcohol consumption during the sub-study period (AOR = 0.69; 95% CI: 0.54, 0.87; p = 0.002) compared to the baseline period. Additionally, female participants were 80% less likely than male individuals to engage in moderate/high risk alcohol intake (AOR = 0.20; 95% CI: 0.12, 0.32; p < 0.001). Conversely, moderate/high-risk alcohol consumption was 2.93 times higher among smokers than among non-smokers (AOR: 2.93; CI: 1.78, 4.85; p < 0.001).

**Table 7:** Output of a Generalized Estimating Equation (GEE) model predicting alcohol consumption risk.

Characteristics	Moderate/high risk alcohol consumption*	
	AOR (95% CI)	*p*-value
Time		
Baseline	1.00	
Sub-study	0.69 (0.54, 0.87)	0.002
Gender		
Male	1.00	
Female	0.20 (0.12, 0.32)	< 0.001
Smoking		
No	1.00	
Yes	2.93 (1.78, 4.85)	< 0.001

*Reference category: None/low risk.

*AOR* adjusted odds ratio.

Table 8 shows the unadjusted cross-sectional association between supplement use and the demographic and health status characteristics. Statistically significant associations were identified between supplements use and only three characteristics at baseline and/or the sub-study. At baseline, a greater percentage of participants who consumed supplements did not have hypertension (p = 0.043) and who had asthma (p = 0.012). At both the baseline and sub-study periods, a greater percentage of participants who consumed supplements were female (p < 0.05).

**Table 8:** Association between supplements use and demographic and health status characteristics.

Characteristics	Supplements use					
	Baseline (2005–2009)			Sub-study (2017)		
	No	Yes	*p*-value	No	Yes	*p*-value
	n (%)	n (%)		n (%)	n (%)	
Gender			< 0.001			0.004
Male	203 (64.2)	113 (35.8)		272 (86.1)	44 (13.9)	
Female	124 (47.7)	136 (52.3)		200 (76.9)	60 (23.1)	
Education			0.685			0.990
No formal school/School only	150 (56.8)	114 (43.2)		227 (82.3)	49 (17.7)	
Trade/apprentice/diploma	113 (58.3)	81 (41.7)		153 (81.8)	34 (18.2)	
University	59 (53.2)	52 (46.8)		89 (82.4)	19 (17.6)	
Marital status			0.515			0.914
Single	25 (56.8)	19 (43.2)		41 (80.4)	10 (19.6)	
Married/living with partner	234 (58.2)	168 (41.8)		297 (82.7)	62 (17.3)	
Widowed/divorced/separated	66 (52.4)	60 (47.6)		130 (82.8)	27 (17.2)	
Smoking			0.080			0.850
No	299 (55.8)	237 (44.2)		399 (81.4)	91 (18.6)	
Yes	28 (70.0)	12 (30.0)		47 (82.5)	10 (17.5)	
Alcohol consumption risk			0.089			0.726
None/low	258 (55.1)	210 (44.9)		395 (81.1)	92 (18.9)	
Moderate/high	62 (64.6)	34 (35.4)		58 (82.9)	12 (17.1)	
BMI (kg/m^2^)			0.926			0.442
Underweight or normal (< 25.0)	108 (58.4)	77 (41.6)		151 (83.9)	29 (16.1)	
Overweight (25.0–29.9)	126 (56.5)	97 (43.5)		154 (79.0)	41 (21.0)	
Obese (≥ 30.0)	70 (56.9)	53 (43.1)		106 (79.7)	27 (20.3)	
Physical activity			0.446			0.998
Inactive/sedentary	78 (59.5)	53 (40.5)		161 (80.9)	38 (19.1)	
Moderate/high	237 (55.8)	188 (44.2)		271 (80.9)	64 (19.1)	
Hypertension			0.043			0.138
No	172 (53.1)	152 (46.9)		313 (83.7)	61 (16.3)	
Yes	155 (61.5)	97 (38.5)		159 (78.7)	43 (21.3)	
Heart disease			0.385			0.627
No	257 (55.9)	203 (44.1)		357 (81.5)	81 (18.5)	
Yes	70 (60.3)	46 (39.7)		115 (83.3)	23 (16.7)	
Diabetes			0.246			0.309
No	278 (55.8)	220 (44.2)		392 (82.7)	82 (17.3)	
Yes	49 (62.8)	29 (37.2)		80 (78.4)	22 (21.6)	
Depression			0.151			0.326
No	243 (56.4)	188 (43.6)		428 (82.5)	91 (17.5)	
Yes	32 (47.1)	36 (52.9)		44 (77.2)	13 (22.8)	
Anxiety			0.292			0.543
No	252 (55.9)	199 (44.1)		431 (82.3)	93 (17.7)	
Yes	23 (47.9)	25 (52.1)		41 (78.9)	11 (21.1)	
Asthma			0.012			0.062
No	250 (57.2)	187 (42.8)		432 (82.9)	89 (17.1)	
Yes	25 (40.3)	37 (59.7)		40 (72.7)	15 (27.3)	

*p*-value is obtained using Chi-square test.

A longitudinal GEE model was used to determine the most important factors for predicting supplement consumption (Table 9). The model shows that participants were 74% less likely to use supplements during the sub-study period (AOR = 0.26; 95% CI: 0.21, 0.34; p < 0.001) compared to the baseline period. Conversely, female participants were 1.83 times more likely than their male counterparts to use supplements (AOR = 1.83; 95% CI: 1.34, 2.49; p < 0.001), while supplements use was 1.71 times higher among participants with asthma (AOR: 1.71; 95% CI: 1.11, 2.62; p = 0.014) compared to those without asthma.

**Table 9:** Output of a Generalized Estimating Equation (GEE) model predicting supplements use.

Characteristics	Supplements use *	*p*-value
	AOR (95% CI)	
Time		
Baseline	1.00	
Sub-study	0.26 (0.21, 0.34)	< 0.001
Gender		
Male	1.00	
Female	1.83 (1.34, 2.49)	< 0.001
Asthma		
No	1.00	
Yes	1.71 (1.11, 2.62)	0.014

*Reference category: no supplements use.

*AOR* adjusted odds ratio.

## Discussion

This is the first large-scale longitudinal analysis focusing upon the determinants of healthy lifestyle behaviours among stroke survivors in NSW, Australia. The study identified a number of interesting and important findings.

Our analysis revealed that moderate/high levels of physical activity were significantly less prevalent during the sub-study period than during the baseline period. These declines in the prevalence of moderate to vigorous physical activity over time may be related to the increased age of stroke survivors, considering that physical fitness and physical function both decline with age, and a number of medical conditions become more prevalent with increasing age^38,39^. Stroke recurrence may also contribute to a decline in physical activity over time, as an estimated 43% of stroke survivors are at risk of having another stroke within ten years of the first stroke^8^, which is associated with a higher risk of physical and mental disability than that associated with the initial stroke^8,40^. Physical inactivity and a sedentary lifestyle can have numerous adverse consequences on the health and well-being of stroke survivors, including an increased risk of disability, physical and cognitive functional decline, the development of comorbid conditions, falls, the onset of mental disorders, and recurrent vascular events^23^. Our finding that the recommended amount of physical activity (≥ 150 min/week) decreases over time is concerning for successful stroke rehabilitation, and it highlights the importance of developing appropriate and effective programmes to promote the recommended amounts of physical activity for all post-stroke individuals throughout their survivorship. Moreover, future research is needed to explore potential strategies for sustaining adherence to the recommended amount of physical activity, with a special focus on elderly stroke survivors.

Another finding from our analysis was that post-stroke individuals who also reported having diabetes were significantly less likely to engage in the recommended amount of physical activity, which is consistent with a longitudinal study conducted in the general population in Germany that showed people with diabetes had lower physical activity levels than those without diabetes^41^. Diabetic patients commonly experience a variety of complications and challenges, including but not limited to depression, coronary heart disease, peripheral vascular disease, chronic kidney disease, liver disease, infection, falls, vision loss, hearing loss, urinary incontinence, cognitive impairment and dementia, frailty, functional disability, and functional limitations^42,43^. These complications may lead to increased levels of disability in stroke survivors who also have diabetes, thereby providing greater challenges for their participation in recommended levels of physical activity. Diabetes is not only a major risk factor for primary and secondary stroke^44^ and a range of complications^42,43^, it is also associated with poor post-stroke recovery^44,45^; and hence, effective diabetes management is crucial in stroke rehabilitation^23,44–46^. Moderate to high levels of physical activity may help stroke survivors with diabetes in reducing their risk of diabetes-related complications and recurrent strokes, as well as enhancing their functional recovery^46^.

The findings of our study also demonstrate a significant association between having a university education and engaging in moderate to high levels of physical activity amongst those living post-stroke. This finding supports previous research among stroke survivors^47^, which demonstrate the importance of high level of education in positively influencing engagement with adequate physical activity. In light of this specific finding from our study further investigation and reflection upon implementing relevant community-based educational programmes may provide one pathway towards facilitating increased adherence to the recommended levels of physical activity among those living post-stroke.

Smoking and alcohol consumption are two leading avoidable causes of premature death and illness^48^. Following a stroke event, modifying health behaviours such as smoking and alcohol consumption is crucial to reduce the risk of developing a variety of illnesses, experiencing worse symptoms, poor functional outcomes, potential drug interactions, and a recurrence of stroke^13^. Several clinical stroke rehabilitation guidelines strongly recommend quitting smoking and reducing alcohol consumption (≤ 14 drinks/week)^9–13^. However, consistent with earlier findings^49^, our study identified that depression was an important predictor for smoking amongst post-stroke individuals. Depression following a stroke is prevalent and can impede the overall process of rehabilitation^50,51^. As depression and smoking are independently associated with stroke recurrence and poor functional outcome among stroke survivors^50–55^, the significant contribution of depression to smoking in our longitudinal study is noteworthy; and our results suggest that effective and appropriate targeted interventions focused upon helping reduce depression following a stroke may be a useful contribution in attempts to reduce smoking prevalence amongst stroke survivors and promote secondary stroke prevention.

Our analyses found that moderate/high-risk alcohol consumption (> 14 drinks/week) among stroke survivors significantly decreased over time. Moderate/high levels of alcohol consumption have several detrimental effects in post-stroke individuals, including a greater risk of developing different medical conditions, having a stroke recurrence, worsening of stroke-related symptoms, interference with certain stroke medications, and poor functional outcomes^56^. Healthcare professionals, friends and family members, community support groups, self-management programmes, and online resources can contribute to the dissemination of information regarding the adverse effects associated with risky alcohol consumption among stroke survivors and serve as sources of motivation to reduce their moderate/high-risk alcohol intake^15^. However, further study is required to determine who and what may be the influencing sources and how we may further encourage and facilitate such sources.

Our study also demonstrated, as expected, a bi-directional relationship between smoking and moderate-high-risk alcohol consumption amongst stroke survivors. The positive relationship between smoking and alcohol consumption is already well-established^57–59^. This association may be due to inter-personal behaviour (i.e., alcohol consumers may also smoke and vice-versa), environmental factors (i.e., users of both substances may use them simultaneously in the same situations), or the fact that alcohol consumption encourages smoking (and vice-versa)^57–59^. As the combined effects of smoking and moderate/high-risk alcohol consumption may have more dangerous health outcomes in post-stroke individuals^13^, the significant link between these behaviours found in our stroke cohort is notable and highlights the importance of potentially ceasing both behaviours to achieve optimal stroke rehabilitation outcomes. Our findings add weight to the need for further investigation of the drivers, enablers and barriers to such behaviours; and consideration of community-based self-management programmes and ongoing social support, amongst other interventions, which may play a substantial role in assisting stroke survivors to quit smoking and reduce the risky alcohol consumption^16,60^.

Our study also identified a significant decline in reported supplement use among stroke survivors over time. However, there is no study in Australia or any other country that is directly comparable to this finding. Possible reasons for the decline in supplement use over time may include the completion of the recommended dosage of the supplements, meeting the desired needs of the users, increased medication burden, potential interaction of supplements with stroke/other medications, and/or failing to experience the expected outcomes from supplement use over time^26,27,61^. Given the potential benefits of dietary supplements for aspects of stroke rehabilitation^26–28^, further research is warranted to investigate various core aspects of supplement use amongst stroke survivors and specifically explore the causes of supplement use decline among stroke survivors with time.

### Limitations

This study has several limitations. The findings of this study, which were based on the stroke survivors of a particular state (NSW), may not be fully generalisable to stroke survivors in other regions of Australia or internationally. The measures included in this study have been widely used and validated in comparable large population samples; nevertheless, they are based on self-report questions that are subject to both recall bias (e.g., incorrectly remembering or misreporting their behaviours) and social desirability bias (e.g., underreporting of socially undesirable habits, such as smoking or excessive alcohol use). Although the 45 and Up Study has a modest baseline response rate of approximately 19%, representativeness is less important in cohort studies, which prioritise internal validity, and the observed associations between cross-sectional exposure and outcomes were comparable to those found in state-based surveillance systems with higher response rates^30^. The robustness of the findings was confirmed by sensitivity analyses, which implies that potential biases had a minimal impact on our study’s outcomes.

Despite encompassing a wide variety of demographic and health-related covariates, the maintenance of healthy lifestyle behaviours may still be influenced by unmeasured confounding factors, such as socioeconomic status, access to healthcare, and the absence of objective measures like wearable devices for physical activity. In addition, diagnostic information to determine whether or not the participants had a stroke, the type of stroke they experienced, and the time since the stroke event was unavailable in the dataset. Future studies incorporating clinical data would help enhance the validity of these findings. Moreover, as our study focused on assessing the independent significant determinants for the outcome variables, the interaction terms between covariates were not incorporated. Future research could investigate these interactions in order to evaluate the combined effects of variables.

Despite these limitations, our study also has several notable strengths, including a large sample size, long-term follow-up and the inclusion of a wide range of demographic and health-related characteristics.

## Conclusion

This study provides the first specific insights into longitudinal determinants of healthy lifestyle behaviours among stroke survivors in NSW, Australia using a large-scale sample with long-term follow-up. The improved understanding about the determinants that significantly contribute to or impede a healthy lifestyle amongst those living post-stroke may help the development of strategies to promote the adoption and maintenance of a healthy lifestyle in stroke survivors as part of their stroke management and rehabilitation, which is crucial to optimising their quality of life and successful secondary stroke prevention. The study suggests some specific groups of post-stroke individuals, such as older adults, individuals with low levels of education, and those diagnosed with diabetes and/or depression, should be the focus of further attention by researchers, policymakers, healthcare professionals, and stroke support groups in order to promote healthy behaviours amongst stroke survivors.

## Data Availability

The data analysed for this study was obtained from the Sax Institute, which coordinates the 45 and Up Study. The data set could potentially be made available to other researchers if they obtain the necessary approvals. Further information on this process can be obtained from the 45 and Up Study (45andUp.research@saxinstitute.org.au).
